# The 5‐year outcomes of a regional population‐based PSA information and testing programme

**DOI:** 10.1111/bju.70180

**Published:** 2026-02-18

**Authors:** Henrik Ugge, Georgios Daouacher, Mauritz Waldén

**Affiliations:** ^1^ Department of Urology, Faculty of Medicine and Health Örebro University Hospital Örebro Sweden; ^2^ School of Medical Sciences Örebro University Örebro Sweden; ^3^ Department of Urology Central Hospital of Karlstad Karlstad Sweden

**Keywords:** prostate cancer, prostate‐specific antigen, screening, testing, population‐based

## Abstract

**Objectives:**

To report descriptive outcomes including prostate‐specific antigen (PSA) testing, diagnostic activity and diagnostic outcomes during the initial 5 years of a regional mailed PSA information and testing programme.

**Subjects and Methods:**

Between 2015 and 2019 a letter regarding the pros and cons of PSA testing was sent to all men in the Värmland region of Sweden in every fifth year age group between 50 and 70 years. A total of 44 530 men were informed and instructed to contact their primary care centre if they wished to have a PSA test. Men with a PSA level ≥3 μg/L were referred to a urologist for evaluation. We used local and national registers to report trends in PSA testing, diagnostic activity, and diagnostic outcomes after programme implementation.

**Results:**

An average of 20% of informed men had a PSA test within 6 months, with increasing proportions over time. Among informed men, 3.5% had a PSA level ≥3 μg/L, 3.6% had a urology consultation, 1.5% underwent biopsies, and 0.8% were diagnosed with prostate cancer. [Correction added on 26 February 2026, after first online publication: The word ‘tested’ has been corrected to ‘informed’ in the preceding sentence.] The proportion who underwent magnetic resonance imaging was low. Between 2014 and 2017, the yearly number of PSA‐tested men in the region increased from 4800 to 11 800 and the number of men undergoing prostate biopsies increased from 772 to 1217. Yearly prostate cancer incidence in the age group 50–74 years increased from 386 (2010–2013) to 659 cases/100 000 (2016–2019). We observed an increase mainly among intermediate‐ and low‐risk tumours. At the end of the period, 80% of men in the older‐age groups had had a PSA test during the preceding years.

**Conclusions:**

Organising a population‐based mailed PSA information programme combined with accessible testing in primary care is feasible on a regional scale in a Scandinavian healthcare setting and may reach high attendance. An observed increase in low‐risk tumours inspires caution against programme implementation and highlights the importance of well‐designed screening strategies and diagnostic algorithms to avoid over‐detection.

**Abbreviations**
ISUP GG: International Society of Urological Pathology Grade Group
NBHW: Swedish National Board of Health and Welfare
NPCR: National Prostate Cancer Register
OPT: Organised Prostate Cancer Testing
PCa: prostate cancer

## Introduction

Prostate cancer (PCa) is the most common form of cancer and the leading cause of cancer‐related death among Swedish men [1, 2]. Early detection of PCa using PSA testing may improve treatment outcomes, and population‐based PSA screening has been observed to reduce PCa mortality in several [3, 4, 5], although not all [6], studies – possibly due to varying degrees of PSA testing in the control population.

Increased detection of clinically insignificant tumours from population‐based PSA screening (over‐detection) increases the risk of treatment‐related adverse effects in the population (overtreatment) [7], calling into question the net benefit of PSA screening for the population. However, recent evidence suggests that modern screening algorithms, using MRI and targeted biopsies, may possibly shift the balance towards a larger net benefit for the population, by reducing detection of low‐risk tumours [8, 9, 10].

In conformity with similar international bodies [11, 12], the Swedish National Board of Health and Welfare (NBHW, Swedish: *Socialstyrelsen*) issued a recommendation in 2014 and again in 2018 advising against population‐based PSA screening based on current evidence [13]. However, problems associated with widespread so‐called opportunistic screening have been acknowledged: a proportion of men in the population undergo PSA testing by patient initiative, often more frequently than required and in a socioeconomically/geographically unequal pattern – likely resulting in a larger problem of overdiagnosis compared to systematic screening, but with less or no mortality benefit in the population [14].

To address this issue, ambitious initiatives regarding organised population‐based PCa testing have been launched in Europe [15]. In Sweden, this has since 2020 taken the form of several regional so‐called ‘Organised Prostate cancer Testing’ (OPT) projects, initiated by the Confederation of Regional Cancer Centres (a collaborative organisation for Swedish healthcare regions), from which initial results have been published [16, 17].

However, already in 2014, the first Swedish national PCa guidelines [18] recommended regional healthcare providers to investigate the effects of systematic information and organised PSA testing. In response to this, the healthcare region of Värmland organised a population‐based PSA information programme, combined with easy access testing in primary care, implemented as a pilot project in 2015, which was later continued and expanded in the form of a healthcare development project. In 2019, the programme had reached all men aged 50–74 years residing in the region. Here, we present descriptive results after the initial 5 years of this programme (2015–2019).

### Objective

To report descriptive results from a regional organised PSA information and testing programme, including PSA testing and diagnostic activity, as well as accompanying trends in PCa diagnostic activity, diagnoses, and treatments on a regional level.

## Subjects and Methods

The Swedish region of Värmland is medium‐sized, sparsely populated, rural, and with an ageing population. The main economy is based on forestry and service businesses. The largest city is the regional capital Karlstad, with ~94 000 inhabitants, while the whole region comprises some 282 000 inhabitants, corresponding to 2.9% of the Swedish population. There were ~46 000 men between the ages of 50 and 74 years at the time of programme implementation. PCa mortality in Värmland has been above the national median, while incidence has been below the national average over the decades until 2014 (local healthcare data), possibly due to sociodemographic factors, which have been demonstrated to influence diagnostic activity [19, 20].

The healthcare system is homogenous and almost exclusively publicly financed. PSA tests are provided at all of the regions’ three hospitals and at ~30 primary care centres but are analysed only at the laboratory at Karlstad Central Hospital. The hospitals and primary care centres share the same computer‐based patient record system, which provides all patient‐related information. While a small minority of the population hold private health insurance and are diagnosed and treated by private healthcare providers throughout Sweden, the overwhelming majority of histologically verified PCas, including information on diagnostics and treatments, are registered in the Swedish National Prostate Cancer Register (NPCR) [21].

Since 2008, the Swedish NBHW has issued a standardised information brochure (available as Data [Link], [Link]) on the pros and cons of PSA testing, recommended for all asymptomatic patients to read before PSA testing in Sweden, in order to promote well‐informed decisions on testing (since 2021 issued by the Confederation of Regional Cancer Centres [22]). As a measure from the regional healthcare authority in Värmland to increase the outreach of information regarding PSA testing in the region, which was considered insufficient at the time, a Working Group was formed in 2013 consisting of local experts – representing urology, primary care, laboratory services and pathology, as well as administrators – who in turn presented a project plan aiming to increase information outreach. By regional political decision, the project started in 2015 as a pilot project, which was subsequently extended.

The Swedish Population Register was used to identify each male individual registered in Värmland. The letters were distributed on a group basis according to birth year: the first year to men becoming 50, 60 and 70 years of age; thereafter to five age groups (50, 55, 60, 65 and 70 years) every year. In the third year the two missing age groups from the first year (by then 57 and 67 years) were added. Men with a previous PCa diagnosis were identified from the Swedish NPCR and excluded. No other information such as recent PSA testing or diagnostic procedures was used to modify the full age group invitation.

Every selected man received a personally addressed letter and the NBHW PSA brochure (translated versions of both available as Data [Link], [Link]), thus being provided information deemed sufficient for a well‐informed decision on PSA testing. The letter stated that both negative and positive aspects of PSA testing among asymptomatic individuals exist and encouraged reading the attached brochure to learn more. The letter further included recommendations for proper testing intervals depending on the PSA result and age, conforming to contemporary national guidelines [18], Finally, the letter stated that if the recipient, after having read the information, wished to have a PSA test, he should contact his primary care centre.

The primary care system was from the start involved in the project and followed a structured procedure: if the patient contacted his primary care centre, an individual evaluation was made by the GP and a PSA test was taken if not deemed inappropriate. When the PSA test was completed, a standardised letter was sent out, which included the result and the recommended year for the next test: if <1 μg/L, a new test was recommended after 6 years; if between 1 and 3 μg/L, a new test was recommended after 2 years, and men aged >60 years with a PSA level <1 μg/L were not recommended further testing in the absence of symptoms. The GPs were educated to follow the testing intervals. Men with an elevated PSA level (≥3 μg/L) were called to the GP and, according to local routine and national guidelines, generally referred to a urologist for further diagnostic evaluation, as part of standard health care. If a PCa diagnosis were eventually to be established, patients would be subjected to appropriate strategies such as curative treatment, palliative treatment, watchful waiting, or active surveillance according to clinical routine. According to Swedish national PCa guidelines at the time, biopsy was indicated in case of suspicious finding on DRE, or a PSA level ≥3 μg/L. Biopsy was not recommended if the PSA level was between 3 and 20 μg/L, with a PSA density <0.1 ng/mL^2^ and a PSA free/total ratio >0.2 [18]. If indication for biopsy was confirmed, guidelines recommended 10–12 biopsies of the lateral parts of the peripheral zone, from apex to base. T‐stage was based on DRE. The systematic use of MRI in primary diagnostic assessment was not established in the region until the very end of the project period, with increased usage from 2019 onward. If indicated based on the urologist's assessment, evaluation of metastases was performed using bone‐scan and/or CT.

The information process started in 2015 and continued for 5 years (2019) until the whole male population in 25 age groups from 50 to 74 years had been informed. A letter was sent to a total of 44 530. The programme is still ongoing, but with substantial changes in the work‐flow over time.

### Outcome Measurements

All clinical procedures and healthcare visits were digitally coded according to the NBHW standard and registered in the digital patient record system (Cambio Cosmic®, Cambio Healthcare Systems, Linköping, Sweden).

All PSA tests in the region from 2004 onwards were registered in the regional laboratory database (CGM Analytix®, CompuGroup Medical [CGM] SE & Co. KGaA, Koblenz, Germany). From that database, the total number of PSA tests and the number of men PSA tested in the primary care for each year during 2010–2019 and the tested proportion of each birth year group (1945–1969) in 2020 was calculated using CGM Explorer® (CGM). Data on performed MRIs were stored in the imaging system Sectra® (Sectra AB, Linköping, Sweden).

Data on the biopsies taken were stored in the register of the regional pathology laboratory (Sympathy®, Tietoevry, Helsinki, Finland). We investigated the number of individuals having a prostate biopsy each year to evaluate the trends.

All healthcare data described above are gathered in the regional healthcare data storage system (‘Datalagret’). This was used to extract group‐level data on numbers and proportions of men per birth‐year group informed who had a PSA test within 6 months of the information letter, PSA level ≥3 μg/L, PSA level <1 μg/L, who had a urology consultation, underwent prostate MRI, underwent biopsies, or were diagnosed with PCa within 9 months of the information letter. To assess PSA testing prior to invitation, we identified PSA tests and biopsies up to 2 years prior to the information letter. We also assessed how many of the men who had a PSA test between 6 months and 2 years after the information letter, and how many who underwent biopsies and received a PCa diagnosis between 9 months and 2 years after the information letter.

The PCa‐risk group distribution was extracted from the NPCR (open data), including all men registered in Region Värmland. As outcome we present the number of men diagnosed with PCa for the Years 2010–2019, categorised by the modified version of D'Amico risk groups recommended by the NBHW and reported in the NPCR [18, 23] at the time into low risk (T1–T2a, International Society of Urological Pathology Grade Group [ISUP GG] 1, and PSA level <10 μg/L), intermediate risk (T2b and/or ISUP GG 2–3 and/or PSA level 10–19.9 μg/L), high risk (T2c–T3 and/or ISUP GG 4–5 and/or PSA level ≥20 μg/L), as well as regional (N1) and distant (M1) metastatic disease.

Treatment information was likewise extracted from the NPCR, including all men settled in the Region Värmland. We present the number of men in different treatment categories for the period 2010–2019, categorised as active surveillance, curative treatment (radical prostatectomy, curative radiotherapy) or non‐curative treatment (conservative treatment, watchful waiting, or palliative treatment). We reviewed registered median waiting times for Karlstad Central Hospital, as well as the national median in the NPCR (time in days from primary care referral to urology visit and time from prostate biopsy to follow‐up visit) to observe trends after project implementation.

We used data from the Swedish Cancer Register [24] to compare PCa incidence during the period 2010–2019 between the Värmland region and the whole of Sweden. The provided incidence was age‐adjusted by weighting according to age‐structure of the Swedish population in 2020 according to the agency's standard. We further compared regional incidences during the period 2010–2013 to those of 2016–2019.

### Ethical Statement

The project was implemented in the context of a politically decided regional healthcare development project, confirmed as such without impediment in an advisory opinion from the Regional Ethical Review Board, Uppsala (Dnr 2015/044). Analysis and presentation of group‐level data from the project was separately approved by the Swedish Ethical Review Authority (Dnr 2024–06013‐01).

## Results

In 2019, 25 birth‐year groups between 50 and 70 at the time of information had received a letter, representing outreach to the whole regional male population aged 50–74 years at the end of the period (44 530 men). Information regarding testing and outcomes per birth‐year group is available in Table 1. The proportion with previous PCa ranged from 6.4% to 10% among 70‐year‐old men (higher proportion later in the period), to 0.1% among 50‐year‐old men. The proportion of men who had a PSA test within 6 months of receiving the letter was >20% for most birth‐year groups aged ≥60 years at time of information and increased later in the period (Table 1); for 50‐year‐old men, the proportion increased from 6.5% to >10% over the period. Men born 1954 and 1959 informed in 2019 (thus aged 65 and 60 years, respectively, at time of information) had a noticeably high proportion of testing (46% and 39%, Table 1). Table S1 shows data grouped by age groups.

**Table 1:** Testing patterns and outcomes per birth‐year group among men aged 50–70 years living in Region Värmland, Sweden, who received mailed PSA information and opportunity to undergo PSA testing in primary care 2015–2019.

Year of information	Year of birth	Men, *n*	Men with previous PCa, *n* (%)*	Men informed, *n*	PSA test within 6 months, *n* (%)^†^	PSA level ≥3 μg/L, *n* (%)^‡^	PSA level <1 μg/L, *n* (%)^‡^	Urology visit, *n* (%)^‡^	Biopsy, *n* (%)^‡^	PCa diagnosis, *n* (%)^‡^
2015	1945	1910	123 (6.4)	1787	407 (22.8)	66 (16.2)	104 (25.6)	61 (15.0)	29 (7.1)	20 (4.9)
2016	1946	1953	149 (7.6)	1804	378 (21.0)	68 (18.0)	99 (26.2)	84 (22.2)	35 (9.3)	23 (6.1)
2017	1947	1867	127 (6.8)	1740	488 (28.0)	84 (17.2)	128 (26.2)	113 (23.2)	38 (7.8)	17 (3.5)
2018	1948	1878	147 (7.8)	1731	409 (23.6)	50 (12.2)	121 (29.6)	84 (20.5)	32 (7.8)	17 (4.2)
2019	1949	1773	179 (10.1)	1594	412 (25.8)	56 (13.6)	131 (31.8)	103 (25.0)	24 (5.8)	14 (3.4)
2017	1950	1785	105 (5.9)	1680	492 (29.3)	144 (29.3)	165 (33.5)	111 (22.6)	49 (10.0)	26 (5.3)
2016	1951	1779	67 (3.8)	1712	344 (20.1)	83 (24.1)	117 (34.0)	71 (20.6)	34 (9.9)	18 (5.2)
2017	1952	1776	73 (4.1)	1703	351 (20.6)	107 (30.5)	112 (31.9)	95 (27.1)	37 (10.5)	16 (4.6)
2018	1953	1776	101 (5.7)	1675	403 (24.1)	124 (30.8)	118 (29.3)	119 (29.5)	26 (6.5)	17 (4.2)
2019	1954	1688	105 (6.2)	1583	728 (45.9)	153 (21)	259 (35.6)	149 (20.5)	71 (9.8)	47 (6.5)
2015	1955	1739	35 (2.0)	1704	274 (16.1)	55 (20.1)	123 (44.9)	28 (10.2)	20 (7.3)	9 (3.3)
2016	1956	1878	40 (2.1)	1838	314 (17.1)	66 (21.0)	125 (39.8)	50 (15.9)	27 (8.6)	11 (3.5)
2017	1957	1779	42 (2.4)	1737	443 (25.5)	83 (18.7)	199 (44.9)	73 (16.5)	36 (8.1)	18 (4.1)
2018	1958	1715	43 (2.5)	1672	337 (20.2)	66 (19.6)	129 (38.3)	63 (18.7)	19 (5.6)	11 (3.3)
2019	1959	1813	47 (2.6)	1766	684 (38.7)	100 (14.6)	312 (45.6)	114 (16.7)	67 (9.8)	24 (3.5)
2017	1960	1705	24 (1.4)	1681	303 (18)	38 (12.5)	161 (53.1)	39 (12.9)	20 (6.6)	7 (2.3)
2016	1961	1717	18 (1.0)	1699	332 (19.5)	29 (8.7)	196 (59.0)	22 (6.6)	10 (3)	7 (2.1)
2017	1962	1813	9 (0.5)	1804	298 (16.5)	28 (9.4)	167 (56.0)	28 (9.4)	13 (4.4)	8 (2.7)
2018	1963	1853	14 (0.8)	1839	210 (11.4)	23 (11.0)	120 (57.1)	28 (13.3)	7 (3.3)	1 (0.5)
2019	1964	2047	21 (1.0)	2026	352 (17.4)	36 (10.2)	188 (53.4)	42 (11.9)	10 (2.8)	4 (1.1)
2015	1965	2065	1 (0.0)	2064	135 (6.5)	10 (7.4)	80 (59.3)	8 (5.9)	4 (3.0)	2 (1.5)
2016	1966	2092	3 (0.1)	2089	261 (12.5)	18 (6.9)	163 (62.5)	19 (7.3)	8 (3.1)	4 (1.5)
2017	1967	2004	4 (0.2)	2000	262 (13.1)	10 (3.8)	166 (63.4)	13 (5.0)	6 (2.3)	0 (0.0)
2018	1968	1838	1 (0.1)	1837	232 (12.6)	12 (5.2)	147 (63.4)	16 (6.9)	1 (0.4)	3 (1.3)
2019	1969	1766	1 (0.1)	1765	192 (10.9)	15 (7.8)	122 (63.5)	17 (8.9)	5 (2.6)	2 (1.0)

* % out of men in birth‐year group.

^†^ % out of informed men.

^‡^ % out of tested men.

Among informed men, 11 528 (25.4%) had had a PSA test in the previous 2 years and 365 (0.8%) had had a biopsy within a year prior to time of the information letter. From 6 months after the invitation up to 2 years after, 7901 men (17.4%) had a PSA test, and from 9 months after the invitation up to 2 years after, 674 (1.5%) had a biopsy and 286 (0.6%) were diagnosed with PCa (data not shown).

The proportion of tested men with a PSA level ≥3 μg/L ranged from 25.6% (467/1826) among 65‐year‐old men, to 6.0% (65/1082) among 50‐year‐old men. The proportion of men with a PSA level <1 μg/L ranged from 27.8% (583/2094) among 70‐year‐old men to 62.7% (678/1082) among 50‐year‐old men. In all, 21.3% (*n* = 445) of 70‐year‐old men and 6.7% (*n* = 73) of 50‐year‐old men were referred to a urologist and prostate biopsies were taken in 7.5% (*n* = 158) of 70‐year‐old men and 2.2% (*n* = 24) of 50‐year‐old men, rendering a PCa diagnosis for 4.3% (*n* = 91) of 70‐year‐old men and 1.0% (*n* = 11) of 50‐year‐old men (Tables 1 and S1). MRI was not used in clinical routine at the start of the period but increased in usage during 2019; around or below 10% of men who had a urology visit during 2015–2018 underwent an MRI, while 40% of men who saw a urologist in 2019 had an MRI (data not shown).

Measures of diagnostic activity in the region are presented in Table 2. The total number of PSA tests in the region increased from 17 064 in 2014 to 26 189 in 2019 (Table 2). This includes all PSA testing in the region including patients with PCa diagnoses. The number of men testing for PSA in the primary care system increased by from 4800 in 2014 to 11 800 in 2017 and thereafter remained on a similar level for the following years (Table 2). The number of individuals undergoing diagnostic biopsies increased from 772 in 2014 to a maximum of 1217 in 2017, and the observed number of detected PCas increased from 369 to 554 in the same period (Table 2). At the start of the programme the proportion of men in the region who had ever had a PSA test during the period for which information was available (from 2004) was 20% among 50‐year‐old men, 43% among 60‐year‐old men, and 63% among 70‐year‐old men (data not shown). In May 2020, the corresponding proportions were 37% among 50‐year‐old men, 67% among 60‐year‐old men, and 76% among 70‐year‐old men (Table S2). The proportion who ever had a PSA test reached >70% in all of the 10 oldest age‐groups (born 1945–1954, Table S2).

**Table 2:** Diagnostic activities in Region Värmland 2010–2019: Total PSA testing (*n*, tests), PSA testing in primary care (*n*, individuals/individuals per 100 000), biopsies (*n*, individuals/biopsied individuals per 100 000) and new PCa cases (*n* diagnoses and age‐adjusted incidence).

Year	Total PSA testing, *n* (tests)	PSA tests in primary care, *n* (individuals)	PSA tests in primary care, tested individuals per 100 000*	Prostate biopsies, *n* (individuals)	Prostate biopsies, individuals per 100 000 population*	New PCa cases, *n*	PCa Incidence, cases per 100 000^†^
2010	17 443	3603	6609	538	987	250	390
2011	16 342	3872	7062	574	1047	290	418
2012	15 603	3934	7127	629	1140	269	398
2013	16 271	4470	8042	585	1052	262	335
2014	17 064	4800	8554	772	1376	369	518
2015	18 899	7451	13 135	853	1504	385	522
2016	21 050	8599	14 993	1030	1796	416	564
2017	24 442	11 800	20 331	1217	2097	544	820
2018	24 630	10 612	18 116	1110	1895	502	667
2019	26 189	10 762	18 238	904	1532	443	585

Implementation of a population‐based PSA information and testing programme commenced in Region Värmland in 2015, reaching full coverage of the male regional population aged 50–74 years in 2019.

* Number of PSA tests and biopsied individuals divided by male regional population aged ≥50 years. Non age‐adjusted due to data availability.

^†^ Incidence from Board of Health and Welfare official statistics, age‐adjusted (standardised) according to age‐structure of the Swedish population in 2020.

**Fig. 1:**
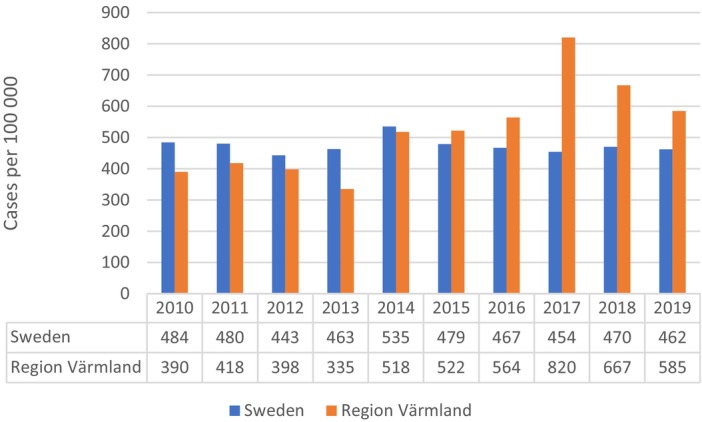
Trends in PCa incidence for the years 2010–2019 among men aged 50–74 years in Sweden and Region Värmland, respectively. Yearly incidence per 100 000, age‐adjusted (weighted) according to the age structure of the Swedish population in 2020.

The observed age‐adjusted incidence of PCa in the age group 50–74 years increased from 386 cases/100 000 during 2010–2013 to 659 cases/100 000 during 2016–2019 (Fig. 1, Table 2), while the national means were 468 and 463 cases/100 000, respectively.

Trends in distribution of new diagnoses in the region by risk category (Table 3) reveal an increase among intermediate‐ and low‐risk PCa during the first years, with some suggestion of a decrease of intermediate‐risk PCa in the last 2 years. At the end of the period, there was a shift in the distribution towards lower‐risk categories, and in 2019 the low‐risk group was the dominating one with 37.5% (compared to 22.4% nationally). The observed yearly number of detected metastatic PCa cases, at the time of diagnosis, did not change markedly over time (Table 3).

**Table 3:** Yearly distribution of new PCa cases for the years 2010–2019 by risk group as registered in the Swedish NPCR, for men living in Sweden and Region Värmland, respectively.

Year of diagnosis	Low risk, *n* (%)	Intermediate risk, *n* (%)	High risk, *n* (%)	Regional metastasis (N1), *n* (%)	Distant metastasis (M1), *n* (%)	Not classified, *n* (%)	Total, *N*
Men living in Region Värmland							
2010	56 (22.4)	70 (28)	66 (26.4)	18 (7.2)	33 (13.2)	7 (2.8)	250
2011	64 (22.1)	86 (29.7)	71 (24.5)	22 (7.6)	42 (14.5)	5 (1.7)	290
2012	65 (24.2)	69 (25.7)	49 (18.2)	23 (8.6)	57 (21.2)	6 (2.2)	269
2013	49 (18.7)	73 (27.9)	61 (23.3)	27 (10.3)	48 (18.3)	4 (1.5)	262
2014	53 (14.4)	112 (30.4)	94 (25.5)	33 (8.9)	61 (16.5)	16 (4.3)	369
2015	105 (27.3)	115 (29.9)	83 (21.6)	29 (7.5)	51 (13.2)	2 (0.5)	385
2016	106 (25.5)	161 (38.7)	86 (20.7)	19 (4.6)	40 (9.6)	4 (1)	416
2017	167 (30.7)	206 (37.9)	80 (14.7)	25 (4.6)	54 (9.9)	12 (2.2)	544
2018	157 (31.3)	179 (35.7)	90 (17.9)	19 (3.8)	42 (8.4)	15 (3)	502
2019	166 (37.5)	147 (33.2)	61 (13.8)	15 (3.4)	43 (9.7)	11 (2.5)	443
Men living in Sweden							
2010	2757 (28.2)	2966 (30.4)	2182 (22.3)	550 (5.6)	1081 (11.1)	230 (2.4)	9766
2011	2697 (28.1)	3036 (31.6)	2096 (21.8)	507 (5.3)	1037 (10.8)	237 (2.5)	9610
2012	2435 (27)	2788 (30.9)	1872 (20.8)	538 (6)	1138 (12.6)	244 (2.7)	9015
2013	2654 (27.6)	3067 (31.9)	1909 (19.9)	532 (5.5)	1219 (12.7)	229 (2.4)	9610
2014	3174 (29)	3584 (32.8)	2098 (19.2)	530 (4.8)	1272 (11.6)	281 (2.6)	10 939
2015	2819 (26.9)	3564 (34)	1992 (19)	589 (5.6)	1261 (12)	247 (2.4)	10 472
2016	2734 (25.7)	3613 (34)	2095 (19.7)	596 (5.6)	1259 (11.9)	323 (3)	10 620
2017	2549 (24.6)	3564 (34.3)	2145 (20.7)	592 (5.7)	1235 (11.9)	294 (2.8)	10 379
2018	2594 (23.7)	4053 (37)	2118 (19.3)	638 (5.8)	1258 (11.5)	288 (2.6)	10 949
2019	2422 (22.4)	4056 (37.4)	2093 (19.3)	557 (5.1)	1246 (11.5)	457 (4.2)	10 831

Implementation of a population‐based PSA‐information and testing programme commenced in Region Värmland in 2015, reaching full coverage of the male regional population aged 50–74 years in 2019.

There was an increase in observed number of men undergoing primary curative treatments during the first years (although noticeably starting already in 2014), with 179–254 patients yearly undergoing treatment after project implementation vs 120–138 patients in the years preceding implementation (Table S3). The number of patients assigned to active surveillance increased dramatically over the period, representing the reported treatment for 34.8% of cases in 2019, compared to ~10% in the years preceding project implementation (Table S3).

The median waiting time at Karlstad Central Hospital, as reported in the NPCR, was longer than the national median at the time of project start (median time from referral to urology visit was 54 days in Karlstad vs 34 days nationally, and time from biopsy to follow‐up visit was 40 days in Karlstad vs 27 days nationally in 2015), but decreased over time both in Värmland and nationally (corresponding figures in 2019 was 16 vs 20 days; and 21 vs 21 days, respectively), with waiting times in Karlstad being close to the national median for most of the period 2016–2019 (data not shown).

## Discussion

Here, we present descriptive outcomes and diagnostic trends 5 years after implementing a regional programme with mailed information on PSA testing combined with accessible testing in primary care, covering the whole male population between 50 and 74 years of age in the Värmland region of Sweden.

Participation rates, measured as PSA tests within 6 months of invitation, was higher in older‐age groups and increased over time for all groups, with a maximum of 46% observed among 65‐year‐old men informed in 2019. Over 5 years, we observed a substantial increase in the number of PSA tests in the region. Using regional and national register data, we further observed an increase in diagnostic activity and in PCa incidence, particularly for low‐ and intermediate‐risk tumours.

As the project was implemented in the form of a healthcare development project, lacking a control group and relying on group‐level healthcare data for some outcome measures, wide‐ranging conclusions on causal associations are inappropriate. However, trends in increased testing and an increasing incidence, particularly for low‐ and intermediate‐risk PCa – in its turn mirrored in an observed higher number of patients undergoing active surveillance and curative treatment – corresponds to expectations [25]. This observation nonetheless warrants caution.

Interestingly, an increase in the reported number of PCas in the region can be observed already in the last year preceding the project (2014), mirrored in a slight increase of PSA tests in primary care, which perhaps reflects increasing awareness of PSA testing over time in the population regardless of the information campaign. The dramatic increase of PSA testing in primary care after project implementation, far exceeding the number of invited men, and the corresponding increase in low‐risk PCa diagnoses, however, seems reasonable to view in the context of direct or indirect effects of the programme. The increase over time in proportion tested within 6 months of the information campaign may suggest an increased awareness in the population over time, possibly as an effect of the information campaign or due to other causes. The observations may hint at unforeseen consequences of the programme, with increased opportunistic testing due to increased awareness in the population – but may also represent secular trends in societal attitudes to PSA testing.

While an increase in testing and diagnostic activity may have put strain on healthcare resources, waiting times for PCa evaluation in the regional urology department did not increase, suggesting an acceptable capacity of the healthcare system manage the consequences of increased testing in the population.

The observed increase in incidence of low‐risk cancer seems to be in an order of magnitude conforming to observations in screening studies [26]. MRI was only used to a limited extent towards the end of the period, and its effects may not yet be visible in the analysed period – although the number of men undergoing biopsies in the region peaked in 2017 while the number of men undergoing PSA testing in primary care continued to increase. The invitation‐selection strategy itself, lacking information of recent PSA testing and diagnostic procedures, may further induce over‐testing or over‐diagnostics. Furthermore, opportunistic repeat testing among invited men may have occurred – as indicated by the fairly substantial proportion who had a PSA test within the 2 years before or after the information letter. This highlights the importance of contemporary screening algorithms including MRI and targeted biopsies, as well as other risk prediction methods and information on previous testing, which may reduce over‐diagnosis [8, 9, 27].

We observed higher proportions of PSA‐tested men in the older‐age groups (corresponding to expectations) up to about 79–80% of 70‐year‐old men. The proportion of tested men increased in all age groups over the period. The proportion of men tested over time was furthermore similar to the proportion tested over long time in screening trials: 77% in the Gothenburg‐1 trial (International Standard Randomised Controlled Trial Number: ISRCTN54449243) [3], 64% (range from 28% in France to 100% in Spain) in the European Randomised Study of Screening for Prostate Cancer (ERSPC) [28]. The observed proportion of 50‐year‐old men who had a PSA test within 6 months of the information letter (11%) was, however, lower than participation rates recently reported from OPT projects in the Swedish metropolitan regions of Stockholm, Gothenburg (Västra Götaland) and Skåne (34–37%) [16]. Initial reports from the southern region of Skåne reported 42% participation (38–45%) among invited men aged 50–62 years. We observed proportions ranging from 11.1% (aged 50 years) to 27.4% (aged 65 years) among men of similar ages. Differences may be explained by geographic differences in socioeconomy affecting attitudes to testing [20], changing attitudes to testing in the population over time, or by differences in invitation/information strategy. However, the observed proportion of 50‐year‐old men in Värmland who had ever had a PSA test (37%) was similar to the reported participation rates from regional OPT‐programmes [16].

One advantage of this report is the large scale, encompassing the entire male regional population aged 50–74 years. Important limitations include lack detailed individual‐level data for important outcomes, such as tumour characteristics, as well as the lack of a comparison group and the present lack of information on repeat testing. However, the Swedish healthcare system and register infrastructure provides the opportunity to review diagnostic activity and characteristics among new cancer cases on a population level. It should further be noted that this project was conducted in a Scandinavian healthcare setting. The information brochure, which was issued by a government agency, proposes to provide balanced information on the pros and cons of PSA testing. The presentation of this information, as well as its reception among readers, may to some extent be culture specific. The degree to which observations can be generalised to other healthcare systems and contexts is unclear.

The project is ongoing. An improved invitation strategy since 2020 involves invitation from a central unit, not involving primary care for testing, as well as repeat testing (interval 2 years if PSA level ≥1 μg/L or missing; 6 years if PSA level <1 μg/L for men aged <65 years). MRI has been established in the routine evaluation of men with PSA levels ≥3 μg/L and the Stockholm3 test (A3P Biomedical AB, Stockholm, Sweden) has been introduced since 2022. The PSA‐information brochure, now published by the Confederation of Regional Cancer Centres, has been updated over time.

In conclusion, the observations in this report support that a population‐based programme providing Swedish men with information about PSA testing, combined with easy access to the test in a distributed primary care organisation, is feasible on a regional level, reaching participation rates over time comparable to previous screening studies. However, signals of increased detection of clinically insignificant PCa conforms to previous observations and may caution against similar programme implementations, particularly in the absence of sophisticated and well‐designed measures aiming to reduce over‐testing and over‐detection, encompassing invitation‐design as well as diagnostic work‐flow.

## Disclosure of Interests

HU has received institutional fees for speaker assignment (Johnson & Johnson). The authors report no other conflicts of interest.

## Supporting information


**Data S1** Letter sent to men aged 50‐70 years living in Värmland Region, translated from the original Swedish by authors.

**Data S2** English translation of the brochure ‘Om PSA‐prov’ (‘About PSA‐testing’).

**Data S3** Brochure ‘Om PSA‐prov' (‘About PSA‐testing'), original Swedish.

**Table S1.** Testing patterns and outcomes per age group among men aged 50–70 years living in Region Värmland, Sweden, who received PSA information and opportunity to undergo PSA testing in primary care 2015–2019.
**Table S2.** Proportion of PSA‐tested men living in Region Värmland in each birth‐year group, defined as ever having had a PSA test during the years 2004–2020.
**Table S3.** Yearly distribution of treatment strategies for new PCa cases diagnosed in Region Värmland in the years 2010–2019, as reported by clinician and registered in the Swedish NPCR.
