# Supplementary material for: The 5‐year outcomes of a regional population‐based PSA information and testing programme
Source: BJU Int. 2026 Feb 18;137(5):877–85. doi: 10.1111/bju.70180 (PMC13071547; doi:10.1111/bju.70180)
Supplement: Supplementary file 1 — Data S1 Letter sent to men aged 50‐70 years living in Värmland Region, translated from the original Swedish by authors. [file BJU-137-877-s003.docx]

**Supplement: Letter sent to men aged 50-70 years living in Värmland Region, translated from the original Swedish by authors.**

**Information about PSA Testing**

With this letter and the enclosed brochure from the National Board of Health and Welfare, the County Council of Värmland wants to inform men between 50 and 70 years of age about the PSA blood test.

It is difficult to know whether the benefits of PSA testing outweigh the negative effects of the test. Therefore, there is no general recommendation that all men should be tested. You decide for yourself whether you want to be tested by weighing the possible advantages and disadvantages of the test.

**Advantages and Disadvantages of PSA Testing**

Prostate cancer detected early can often be cured. The simplest way to detect prostate cancer is with a simple PSA blood test. The main advantage of the PSA test is that early detection and treatment of prostate cancer reduces the risk of serious prostate cancer in the future.

The main disadvantage is that some men may be diagnosed with prostate cancer and possibly receive treatment even though the cancer would never have developed into a serious disease. More men are treated after PSA testing than are cured of life-threatening cancer. The treatment can cause lasting side effects.

**Read the Brochure and Decide Whether You Want to Be Tested**

Read the enclosed brochure for more information. If, after reading the brochure, you want to be tested, you can contact your health center to have the PSA blood test taken. If you have further questions or concerns after reading the brochure, you are welcome to contact our PSA nurse at telephone number 054–61 44 80, Monday–Thursday, 1:00–4:00 PM.

More information about the PSA test is also available at:
http://www.1177.se/Varmland/Fakta-och-rad/Undersokningar/PSA/

Have you already been tested? Turn the page and read about when a new test might be relevant for you.

**Best regards,**
Mauritz Waldén
Senior Physician, Urology Section, Surgical Clinic, Central Hospital in Karlstad
County Council of Värmland

**Intervals for PSA Testing**

If a PSA value below 1.0 has been found, the next test is not needed until after six years.
Men over 60 years of age with a PSA value below 1.0 do not need any further PSA blood tests.
Men with a PSA value between 1 and 3 should be checked every other year. Your doctor will inform you about what applies to you. Also tell your doctor if at least two close relatives have had prostate cancer, as regular check-ups are then recommended.

**NOTE:** More frequent testing than the above provides no benefit at all! Avoid unnecessary testing!
