# Supplementary material for: The 5‐year outcomes of a regional population‐based PSA information and testing programme
Source: BJU Int. 2026 Feb 18;137(5):877–85. doi: 10.1111/bju.70180 (PMC13071547; doi:10.1111/bju.70180)
Supplement: Supplementary file 2 — Data S2 English translation of the brochure ‘Om PSA‐prov’ (‘About PSA‐testing’). [file BJU-137-877-s004.docx]

**Supplement: English translation of the brochure “Om PSA-prov” (“About PSA-testing”)**

Issued by the Swedish National Board of Health and Welfare (NBHW) 2014-08-14, translated using Microsoft Copilot and reviewed by authors, for publication as supplementary material in BUJI, with permission by correspondence from NBHW.

**Should Men Have Prostate Checks?**

For most types of cancer, it is almost always beneficial for the cancer to be detected and treated before it causes any symptoms. That is why regular screenings are offered to detect early forms of, for example, breast cancer and cervical cancer.

However, it is not certain that the benefits of screening for prostate cancer outweigh the disadvantages for those who have no symptoms. Therefore, prostate checks are not offered routinely. Instead, men themselves must decide whether they want to undergo an examination after personally weighing the pros and cons. This brochure can provide a basis for such an evaluation.

Men who have urinary symptoms should always contact a doctor.

**The Most Important Pros and Cons of the PSA Test**

The main advantage of the PSA test is that early detection and treatment can reduce the risk of serious prostate cancer in the future.

The main disadvantage is that many men are diagnosed with prostate cancer and receive treatment even though the cancer would never have developed into a serious disease. Far more men are treated unnecessarily after a PSA test than are cured of life-threatening cancer. Treatment often leads to lasting side effects.

If 1,000 men aged 50–70 choose not to have PSA testing, 9 of them will die from prostate cancer within 14 years. If these men instead had regular PSA tests, only 5 of them would die from prostate cancer in the same period. At the same time, PSA testing would lead to 50 of the tested men being diagnosed with prostate cancer and possibly treated, even though they would never have developed serious prostate cancer.

**Men Decide for Themselves Whether to Be Tested**

The National Board of Health and Welfare has assessed that the benefits of general PSA testing do not outweigh the disadvantages from a population perspective. However, as an individual, you may weigh the potential benefits and disadvantages differently. Men who have received information about the pros and cons of PSA testing and who then wish to undergo testing should be allowed to do so.

One argument in favor of regular PSA testing is having several relatives who have had prostate cancer, as this increases the risk of developing the disease yourself.

If avoiding repeated examinations and treatment side effects feels important, that may argue against PSA testing. Men with another disease that could become serious within the next few years have very little chance of benefiting from PSA testing, as it usually takes more than 10 years for a small prostate cancer to develop into a serious illness.

**What Is the Prostate Gland?**

The prostate gland surrounds the urethra just below the bladder. It is needed for reproduction. From middle age, the prostate gland enlarges in most men, a condition called benign prostate enlargement. Cancer in the prostate gland also becomes more common with age, but there is no link between benign enlargement and prostate cancer. Both benign enlargement and prostate cancer can cause a weaker urine stream and more frequent urination.

**Prostate Cancer**

Prostate cancer is the most common cancer in Sweden. Most people who develop prostate cancer are over 70 years old, and the disease is rare before age 50. The risk increases if you have close relatives who have had the disease. One in twenty Swedish men dies from prostate cancer, and half of them are over 80 years old.

When prostate cancer causes clear symptoms, it can rarely be cured, but the disease can be alleviated and slowed with hormonal treatment. Urinary problems and pain are common symptoms.

Although prostate cancer is often a serious disease, most cancerous areas in the prostate gland are small and mild. They grow so slowly that a man usually dies of something else before it becomes a large tumor. Many more men die with prostate cancer than from it.

**Prostate Check with PSA Test to Detect Cancer**

PSA stands for prostate-specific antigen. PSA is a substance produced in the prostate gland and secreted in semen. All prostate diseases can lead to increased PSA levels in the blood. An elevated PSA level in a blood test does not necessarily mean you have cancer.

**At What Ages Can PSA Testing Be Relevant?**

Prostate cancer is rare before age 50. In men over 75, a small prostate cancer rarely develops into a serious disease. Therefore, PSA testing may be relevant for men aged 50 to 75 who have no symptoms of prostate cancer.

**Most Men Have Low PSA Levels**

Most men have low PSA levels in the blood, but the value increases with age. Men with low PSA levels have a very small risk of prostate cancer requiring treatment. If you have a low PSA level, it may be appropriate to repeat the PSA test after 2–6 years, depending on how low the value was.

**What Does an Elevated PSA Level Mean?**

One in ten middle-aged men has an elevated PSA level. With repeated testing over many years, one in four men will have an elevated PSA level.

Men with PSA levels above a certain threshold usually undergo further examinations to determine whether there is cancer in the prostate gland. A doctor then examines the prostate gland with a finger in the rectum. Usually, an ultrasound examination via the rectum is also performed, along with tissue sampling from the prostate gland. These examinations may feel uncomfortable but are usually not painful.

Tissue samples show cancer in one-quarter of men with moderately elevated PSA levels. Many of the cancerous areas detected are small and would never have caused illness. If a more serious cancer is found after a PSA test, it is usually detected 5–15 years before it would have caused symptoms.

Most men with moderately elevated PSA levels do not have prostate cancer. The cause of the PSA increase is usually benign prostate enlargement. However, it is difficult to be completely sure that there is no prostate cancer in a man with elevated PSA levels. Therefore, repeated tissue sampling is common in men with elevated PSA, even if they are actually healthy. For men with elevated PSA levels, the uncertainty about whether they have cancer can be worrying.

**Treatment of Early Prostate Cancer**

Prostate cancer detected after a PSA test can usually be cured with either surgery or radiation therapy. Both treatments often lead to reduced erectile function (difficulty achieving an erection). Radiation therapy sometimes causes rectal problems and can increase the need to urinate. Surgery can sometimes cause troublesome urinary leakage.

More than one-third of men diagnosed with prostate cancer after a PSA test have a small cancer that looks mild under the microscope. In such cases, the usual approach is to monitor the development with regular check-ups instead of treating immediately.

**How to Learn More?**

There is no rush to make a decision. You can read more detailed information at www.1177.se. You can also get help from your general practitioner to weigh the pros and cons.
