# Supplementary material for: The 5‐year outcomes of a regional population‐based PSA information and testing programme
Source: BJU Int. 2026 Feb 18;137(5):877–85. doi: 10.1111/bju.70180 (PMC13071547; doi:10.1111/bju.70180)
Supplement: Supplementary file 3 — Data S3 Brochure ‘Om PSA‐prov' (‘About PSA‐testing'), original Swedish. [file BJU-137-877-s002.pdf]

Socialstyrelsen

Om PSA-prov

För att kunna upptäcka prostatacancer
i ett tidigt skede – fördelar och nackdelar

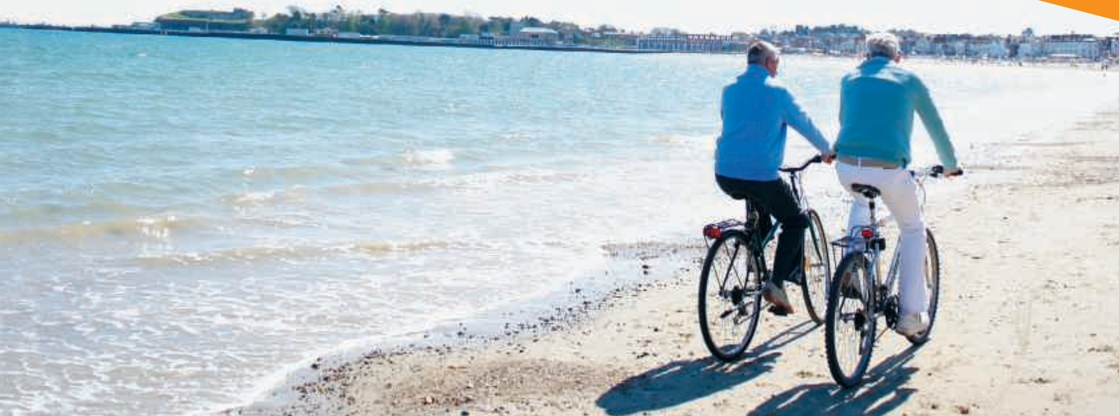

Information från Socialstyrelsen

Augusti 2014. Detta är en uppdaterad version av de broschyrer som utkom 2007 och 2010.

Samverkan med andra aktörer

Regionala cancercentrum i samverkan

Svensk urologisk förening

Svensk onkologisk förening

Svensk förening för allmänmedicin

Svenska företagsläkarföreningen

Prostatacancerförbundet

Du får gärna citera Socialstyrelsens texter om du uppger källan, exempelvis i utbildningsmaterial till självkostnadspris, men du får inte använda texterna i kommersiella sammanhang. Socialstyrelsen har ensamrätt att bestämma hur detta verk får användas, enligt lagen (1960:729) om upphovsrätt till litterära och konstnärliga verk (upphovsrättslagen). Även bilder, fotografier och illustrationer är skyddade av upphovsrätten, och du måste ha upphovsmannens tillstånd för att använda dem.

ISBN 978-91-7555-201-9
Artikelnr 2014-8-4

Foto AgeFotostock
Illustration Kari Toverud
Tryck Edita Bobergs AB, Falun, augusti 2014

MILJÖMÄRKT TRYCKSAK 341 009

Ska män göra prostatakontroller?

Vid de flesta cancerformer är det nästan alltid bra att cancern upptäcks och behandlas innan den ger några symtom. Därför erbjuds regelbundna undersökningar för att upptäcka tidiga former av till exempel bröstcancer och livmoderhalscancer.

Det är däremot inte säkert att fördelarna med en undersökning för att upptäcka prostatacancer överväger nackdelarna för dem som inte har några symtom. Prostatakontroller erbjuds därför inte rutinmässigt. I stället får män själva avgöra om de vill göra en undersökning efter en personlig värdering av fördelar och nackdelar. Denna broschyr kan ge underlag för en sådan värdering.

Män som har symtom från urinvägarna bör alltid kontakta läkare.

De viktigaste fördelarna och nackdelarna med PSA-prov

Den viktigaste fördelen med PSA-prov är att tidig upptäckt och behandling kan minska risken för allvarlig prostatacancer i framtiden.

Den viktigaste nackdelen är att många män diagnostiseras med prostatacancer och får behandling trots att cancern aldrig skulle ha utvecklats till en allvarlig sjukdom. Det är betydligt fler män som behandlas i onödan efter PSA-prov, än som botas från en livshotande cancer. Behandlingen ger ofta bestående biverkningar.

Om 1 000 män i åldern 50–70 år låter bli att PSA-testas kommer 9 av dem att dö av prostatacancer inom 14 år. Om dessa män i stället hade PSA-testats regelbundet kommer bara 5 av dem dö av prostatacancer inom samma tid. Samtidigt skulle PSA-testningen leda till att 50 av de testade männen får en prostatacancerdiagnos och kanske även behandling, trots att de aldrig skulle ha utvecklat en allvarlig prostatacancer.

Män får själva ta ställning till om de vill testa sig

Socialstyrelsen har bedömt att fördelarna med allmänna PSA-prov inte uppväger nackdelarna i ett befolkningsperspektiv. Som enskild individ kan man dock värdera de tänkbara fördelarna och nackdelarna på ett annat sätt. Män som har tagit del av information om för- och nackdelarna med PSA-prov och som därefter önskar genomgå provtagning bör därför få göra detta.

Något som talar för regelbundna PSA-prov är om man har flera släktingar som drabbats av prostatacancer, eftersom risken då är större att man själv ska få sjukdomen.

Om det känns viktigt att undvika upprepade undersökningar och biverkningar av behandlingar, kan det tala emot PSA-prov. Män som har någon annan sjukdom som kan bli allvarlig inom de närmaste åren har mycket liten chans att ha nytta av PSA-prov, eftersom det vanligen tar mer än 10 år för en liten prostatacancer att utvecklas till en allvarlig sjukdom.

Vad är prostatakörteln?

Prostatakörteln omsluter urinröret precis nedanför urinblåsan. Den behövs för fortplantingen. Från medelåldern växer prostatakörteln hos de flesta män, så kallad godartad prostataförstoring. Cancer i prostatakörteln blir också vanligare med stigande ålder, men det finns inget samband mellan godartad förstoring och prostatacancer. Både godartad prostataförstoring och prostatacancer kan medföra att urinstrålen blir svagare och att man kan få gå och kissa oftare.

Prostatacancer

Prostatacancer är den vanligaste cancersjukdomen i Sverige. De flesta som insjuknar i prostatacancer är över 70 år och sjukdomen är sällsynt före 50 års ålder. Risken att drabbas ökar om man har nära släktingar som har haft sjukdomen. Var tjugonde svensk man dör av prostatacancer. Hälften är då över 80 år gamla.

När prostatacancer ger tydliga symtom kan den sällan botas, men sjukdomen kan lindras och bromsas med hormonell behandling. Urinvägsbesvär och smärtor är vanliga symtom.

Trots att prostatacancer ofta är en allvarlig sjukdom, är de flesta härdar av cancerceller i prostatakörteln små och beskedliga. De växer så långsamt att mannen hinner dö av någonting annat innan det blir en stor cancertumör i prostatakörteln. Många fler män dör *med* än *av* prostatacancer.

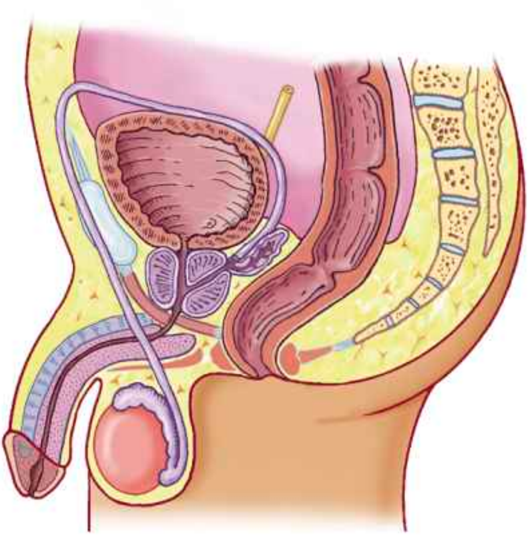

Prostatakontroll med PSA-prov för att upptäcka cancer

PSA betyder prostataspecifikt antigen. PSA är ett ämne som produceras i prostatakörteln och utsöndras i sädesvätskan. Alla sjukdomar i prostatakörteln kan leda till att halten av PSA i blodet ökar. Ett förhöjt PSA-värde i ett blodprov behöver alltså inte betyda att man har cancer.

I vilka åldrar kan PSA-prov vara aktuellt?

Prostatacancer är sällsynt före 50 års ålder. Hos män över 75 år hinner en liten prostatacancer sällan utvecklas till någon allvarlig sjukdom. Därför är det mellan 50 och 75 års ålder som PSA-prov kan vara aktuellt för män utan symtom av prostatacancer.

De flesta har ett lågt PSA-värde

De allra flesta män har låga halter av PSA i blodet, men värdet ökar med åldern. Män med låga PSA-värden har mycket liten risk för behandlingskrävande prostatacancer. Om man har ett lågt PSA-värde kan det vara lämpligt att upprepa PSA-provet efter 2–6 år, beroende på hur lågt värdet var.

Vad innebär ett förhöjt PSA-värde?

Var tionde medelålders man har förhöjt PSA-värde. Vid upprepad provtagning under många år kommer var fjärde man att ha ett förhöjt PSA-värde.

Hos män med PSA-värden över en viss nivå brukar man göra ytterligare undersökningar för att ta reda på om det finns cancer i prostatakörteln. En läkare undersöker då prostatakörteln med ett finger i ändtarmen. För det mesta görs också en ultraljudsundersökning via ändtarmen med samtidig vävnadsprovtagning från prostatakörteln. Undersökningarna kan upplevas som obehagliga, men är vanligen inte smärtsamma.

Vävnadsproven visar cancer hos en fjärdedel av männen med ett måttligt förhöjt PSA-värde. Många av de cancerhärdar som upptäcks är små och skulle aldrig ha lett till någon sjukdom. Om man hittar en mer allvarlig cancer efter ett PSA-prov, brukar den upptäckas 5–15 år innan den skulle ha gett symtom.

De flesta som har ett måttligt förhöjt PSA-värde har alltså inte prostatacancer. Orsaken till PSA-ökningen är i så fall vanligen en

godartad prostataförstoring. Det är dock svårt att vara helt säker på att det inte finns någon prostatacancer hos en man med förhöjt PSA-värde. Därför är det vanligt att man får göra upprepade vävnadsprov om man har förhöjt PSA, fast man egentligen är frisk. För män med förhöjda PSA-värden kan osäkerheten om man har cancer eller inte vara oroande.

Behandling av tidig prostatacancer

Prostatacancer som upptäcks efter ett PSA-prov kan vanligen botas med antingen operation eller strålbehandling. Båda dessa behandlingar leder oftast till försämrad erektionsförmåga (svårt att få stånd). Strålbehandlingen ger ibland ändtarmsbesvär och kan göra att man får behov att kissa oftare. Operationen kan ibland medföra besvärande urinläckage.

Mer än en tredjedel av de män som diagnostiseras med prostatacancer efter PSA-prov har en liten cancer som ser beskedlig ut i mikroskop. Då brukar man följa utvecklingen med regelbundna kontroller i stället för att behandla direkt.

Hur får man veta mer?

Att fatta beslut är inget som brådskar. Du kan läsa mer detaljerad information på www.1177.se. Du kan också få hjälp av din allmänläkare att väga för- och nackdelar mot varandra.

Hur gör man om man vill testa sig?

Efter att ha diskuterat med din läkare har du möjlighet att göra en prostatakontroll med PSA-prov. Den läkare som har ordinerat provet ger dig sedan besked om resultatet och om vad det innebär.

Denna broschyr innehåller information för män som funderar på att göra en hälsokontroll med PSA-prov för att minska sin risk att drabbas av allvarlig prostatacancer. Broschyren beskriver de fördelar och nackdelar som finns med PSA-prov, och ger underlag för en personlig värdering av dessa.

Enligt Socialstyrelsens nationella riktlinjer från 2014 bör symtomfria män inte PSA-testas utan att de först har tagit del av informationen i denna broschyr. Detta är en uppdatering av de broschyrer som publicerades 2007 och 2010.

Vill du veta mer om de nationella riktlinjerna för prostatacancer kan du ladda ner eller beställa dessa från Socialstyrelsens webbplats, www.socialstyrelsen.se/nationellariktlinjer.

Om PSA-prov
(artnr 2014-8-4) kan beställas från
Socialstyrelsens publikationsservice.
www.socialstyrelsen.se/publikationer
E-post: publikationsservice@socialstyrelsen.se
Fax: 035-19 75 29

Publikationen kan även laddas ner från
www.socialstyrelsen.se

Socialstyrelsen
