# Supplementary material for: The 5‐year outcomes of a regional population‐based PSA information and testing programme
Source: BJU Int. 2026 Feb 18;137(5):877–85. doi: 10.1111/bju.70180 (PMC13071547; doi:10.1111/bju.70180)
Supplement: Supplementary file 4 — Table S1. Testing patterns and outcomes per age group among men aged 50–70 years living in Region Värmland, Sweden, who received PSA information and opportunity to undergo PSA testing in primary care 2015–2019. Table S2. Proportion of PSA‐tested men living in Region Värmland in each birth‐year group, defined as ever having had a PSA test during the years 2004–2020. Table S3. Yearly distribution of treatment strategies for new PCa cases diagnosed in Region Värmland in the years 2010–2019, as reported by clinician and registered in the Swedish NPCR. [file BJU-137-877-s001.docx]

**Supplementary Table 1**: Testing patterns and outcomes per age group among men aged 50-70 years living in Region Värmland, Sweden, who received PSA-information and opportunity to undergo PSA-testing in primary care 2015-2019.

| Age group, years | Men informed, N | PSA within 6 mo, n (%)^a^ | PSA ≥3 µg/L, n (%)^b^ | PSA <1µg/L, n (%)^b^ | Urology visit, n (%)^b^ | Biopsy, n (%)^b^ | PCa diagnosis, n (%)^b^ |
| --- | --- | --- | --- | --- | --- | --- | --- |
|  |  |  |  |  |  |  |  |
| 70 | 8656 | 2094 (24,2) | 324 (15,5) | 583 (27,8) | 445 (21,3) | 158 (7,5) | 91 (4,3) |
| 67^c^ | 1680 | 492 (29,3) | 144 (29,3) | 165 (33,5) | 111 (22,6) | 49 (10.0) | 26 (5,3) |
| 65 | 6673 | 1826 (27,4) | 467 (25,6) | 606 (33,2) | 434 (23,8) | 168 (9,2) | 98 (5,4) |
| 60 | 8717 | 2052 (23,5) | 370 (18.0) | 888 (43,3) | 328 (16.0) | 169 (8,2) | 73 (3,6) |
| 57^c^ | 1681 | 303 (18.0) | 38 (12,5) | 161 (53,1) | 39 (12,9) | 20 (6,6) | 7 (2,3) |
| 55 | 7368 | 1192 (16,2) | 116 (9,7) | 671 (56,3) | 120 (10,1) | 40 (3,4) | 20 (1,7) |
| 50 | 9755 | 1082 (11,1) | 65 (6.0) | 678 (62,7) | 73 (6,7) | 24 (2,2) | 11 (1.0) |
| a: % out of informed men. b: % out of tested men. c: previously non-invited birthyear-groups invited in 2017 | | | | | | | |

**Supplementary Table 2**: Proportion of PSA tested men living in Region Värmland in each birth year group, defined as ever having had a PSA-test during the years 2004-2020. Implementation of a population-based PSA-information programme commenced in Region Värmland in 2015, reaching full coverage of the male regional population aged 50-74 years in 2019.

| Year of birth | PSA tested (%) |
| --- | --- |
| 1945 | 80% |
| 1946 | 80% |
| 1947 | 80% |
| 1948 | 78% |
| 1949 | 76% |
| 1950 | 80% |
| 1951 | 74% |
| 1952 | 74% |
| 1953 | 73% |
| 1954 | 76% |
| 1955 | 66% |
| 1956 | 65% |
| 1957 | 67% |
| 1958 | 61% |
| 1959 | 67% |
| 1960 | 57% |
| 1961 | 59% |
| 1962 | 56% |
| 1963 | 50% |
| 1964 | 50% |
| 1965 | 55% |
| 1966 | 45% |
| 1967 | 45% |
| 1968 | 40% |
| 1969 | 37% |
| Total | 64% |

**Supplementary Table 3**: Yearly distribution of treatment strategies for new prostate cancer cases diagnosed in Region Värmland in the years 2010-2019, as reported by clinician and registered in the Swedish National Prostate Cancer Register (NPCR). Implementation of a population-based PSA-information programme commenced in Region Värmland in 2015, reaching full coverage of the male regional population aged 50-74 years in 2019.

| Year of diagnosis | Treatment strategy, n (%) | | | | |
| --- | --- | --- | --- | --- | --- |
|  | Active surveillance | Curative^a^ | Non-curative^b^ | Unknown | Total |
| 2010 | 29 (11,6) | 120 (48.0) | 101 (40,4) | 0 (0.0) | 250 |
| 2011 | 30 (10,3) | 136 (46,9) | 123 (42,4) | 1 (0,3) | 290 |
| 2012 | 24 (8,9) | 136 (50,5) | 108 (40,1) | 1 (0,4) | 269 |
| 2013 | 21 (8.0) | 138 (52,7) | 102 (38,9) | 1 (0,4) | 262 |
| 2014 | 25 (6,8) | 204 (55,3) | 139 (37,7) | 1 (0,3) | 369 |
| 2015 | 66 (17,1) | 195 (50,7) | 123 (32.0) | 1 (0,3) | 385 |
| 2016 | 85 (20,4) | 210 (50,5) | 116 (27,9) | 5 (1,2) | 416 |
| 2017 | 166 (30,5) | 254 (46,7) | 113 (20,8) | 11 (2.0) | 544 |
| 2018 | 157 (31,3) | 203 (40,4) | 133 (26,5) | 9 (1,8) | 502 |
| 2019 | 154 (34,8) | 179 (40,4) | 105 (23,7) | 5 (1,1) | 443 |
| a Including curative radiotherapy and radical prostatectomy | | | | | |
| b Including watchful waiting and palliative treatment strategies | | | | |  |
